# Awareness, access, and communication: provider perspectives on early intervention services for children with sickle cell disease

**DOI:** 10.3389/fped.2024.1366522

**Published:** 2024-03-25

**Authors:** Andrew M. Heitzer, Erin MacArthur, Mollie Tamboli, Ashley Wilson, Jane S. Hankins, Catherine R. Hoyt

**Affiliations:** ^1^ Department of Psychology and Biobehavioral Sciences, St. Jude Children’s Research Hospital, Memphis, TN, United States; ^2^ Program in Occupational Therapy, Washington University in St. Louis, St. Louis, MO, United States; ^3^ Department of Global Pediatric Medicine, St. Jude Children’s Research Hospital, Memphis, TN, United States; ^4^ Department of Hematology, St. Jude Children’s Research Hospital, Memphis, TN, United States; ^5^ Program in Occupational Therapy, Departments of Neurology and Pediatrics, Washington University in St. Louis, St. Louis, MO, United States

**Keywords:** sickle cell, early intervention, developmental, cognitive, occupational therapy, physical therapy, speech therapy

## Abstract

**Purpose:**

This study aimed to identify determinants influencing the utilization of early intervention services among young children with sickle cell disease (SCD) based on perspectives from medical and early intervention providers.

**Design and methods:**

Early intervention and medical providers from the catchment area surrounding St. Jude Children's Research Hospital and Washington University were recruited (20 total providers). Interviews were completed over the phone and audio recorded. All interviews were transcribed verbatim, coded, and analyzed using inductive thematic analysis.

**Results:**

Three overarching themes were identified from both groups: Awareness (e.g., lack of awareness about the EI system and SCD), Access (e.g., difficulties accessing services), and Communication (e.g., limited communication between medical and early intervention providers, and between providers and families). Although these three themes were shared by medical and early intervention providers, the differing perspectives of each produced subthemes unique to the two professional fields.

**Conclusions:**

Early intervention services can limit the neurodevelopmental deficits experienced by young children with SCD; however, most children with SCD do not receive these services. The perspectives of early intervention and medical providers highlight several potential solutions to increase early intervention utilization among young children with SCD.

## Introduction

Sickle cell disease (SCD) is a monogenic blood disorder of growing global health concern affecting approximately 300,000 people, mostly in sub-Saharan Africa, the Middle East, and India (1, 2). In the United States, approximately 100,000 people have SCD; the majority of whom are Black or of African descent. This population has been historically excluded from high-quality health care and are often living in low-income, underserved communities (3, 4). Due to mandated newborn screening and penicillin prophylaxis, early mortality has substantially reduced; however, disease complications significantly interfere with quality of life across the lifespan (5–7). Notably, SCD, with and without stroke, is characterized by early and progressive neurocognitive deficits associated with poor school readiness (8), grade retention (9), and unemployment (10). Early Intervention (EI) is a nationwide program that can improve neurodevelopmental outcomes (11, 12), but many families do not utilize these services.

Due to early neurodevelopmental risk and the potential of EI to capitalize on neural plasticity, it is important to conduct neurodevelopmental screenings among children with SCD at the earliest possible age to identify patients requiring treatment. Once developmental concerns are identified, implementing evidence-based EI services is imperative. EI encompasses a variety of services, most often including physical, occupational, and speech therapy. Prior studies have demonstrated that home-based caregiver-driven interventions can ameliorate the impact of developmental deficits among children with SCD (13). Yet, many barriers exist for families attempting to obtain EI. Available evidence suggests that very few children with SCD are successfully referred to and utilize intervention resources (14). SCD is not a qualifying diagnosis for EI in most states, and signs of developmental delays in children with SCD are often missed due to their subtle nature early in life (15, 16). Further, due to systemic racism and inequities, Black children are less likely to receive EI services than white children if they do not automatically qualify based on a medical condition (17).

Prior studies of neurobehavioral intervention in SCD have been hindered by poor adherence (13, 18, 19). To increase acceptability and adherence to evidence-based intervention services, it is necessary to identify the determinants that influence program participation for families with children identified to be at risk. Prior studies among low-income African-American and Hispanic families have highlighted that caregiver distrust, conflicting information from providers, and competing stressors (e.g., work, childcare, etc.) have interfered with EI utilization (20).

Prior qualitative investigations with EI providers have highlighted the discrepancies in provider perspectives compared to established best practices in EI (21). These investigations provided insight into changes that were needed to improve intervention adherence and provider satisfaction. Focus groups with caregivers, pediatricians, and EI service providers led to recommendations for communication training, skill building, and the utilization of technology to enhance communication (22). Due to the unique sociodemographic characteristics and medical needs of the SCD population, it is important to understand both medical and EI provider perspectives on how to best facilitate EI services for this population. Prior qualitative investigations by our group with EI providers and caregivers highlighted the need for EI screening and referral for children with SCD but noted that incentives are needed to encourage providers to change current practice patterns (23).

The objective of the current study was to identify determinants influencing EI utilization among young children with SCD. We used qualitative methods to describe EI and medical providers' current practices and to characterize challenges and strategies for overcoming obstacles at two sites in the United States. The study aimed to assess provider perspectives as the next steps toward designing implementation strategies to improve EI access for young children with SCD.

## Methods

The Institutional Review Boards approved study procedures at both participating sites.

### Participants

EI and medical providers from the area surrounding St. Jude Children's Research Hospital and Washington University were recruited. Each site recruited five EI providers and five medical providers. The sample size (*n* = 20) was determined prior to recruitment based on prior qualitative research as well as the relatively narrow aims of the research and highly specific sample (24).

Principal investigators contacted colleagues who may be interested in participating. Following the initial contacts, a snowball sampling method was used, with initial contacts providing additional providers who may be interested in participating. The sample of medical providers included pediatric primary care physicians (*n* = 3), pediatric hematologists (*n* = 2), hematology nurse practitioners (*n* = 4), and a social worker (*n* = 1). The EI sample was predominantly occupational therapists (*n* = 7), including one physical therapist, parent educator, and assessment specialist. The EI providers predominately practice in the families' homes or daycare centers.

### Data collection and analysis

Semi-structured interviews were developed using the Reach, Effectiveness, Adoption, Implementation, and Maintenance (RE-AIM) framework to evaluate factors influencing early intervention program utilization (25). The interview guide was developed by the research team and reviewed by content experts. Each interview prompt was mapped to a construct of RE-AIM (see Supplementary Files for Interview Guides). Interviews were completed over the phone and audio recorded. All interviews were transcribed verbatim and reviewed by two team members at each site for accuracy. Two research team members used an iterative process to review all qualitative transcripts using DelveTool Software (26). Coders used an inductive thematic analysis approach to generate themes for a preliminary codebook (27). Separate codebooks were created for the medical providers and the EI providers. Two EI provider interviews and two medical provider interviews, one of each from both sites, were reviewed to develop preliminary codes. Any deviations or challenges were discussed, and codes were revised into the final versions of the codebooks. The medical provider codebook consisted of 16 codes, and the EI provider codebook included 15 codes. The revised codebook was applied to all interviews. The codes and corresponding quotes were reviewed and coalesced into the main themes that were identified in the majority of the interviews.

## Results

**Figure 1:**
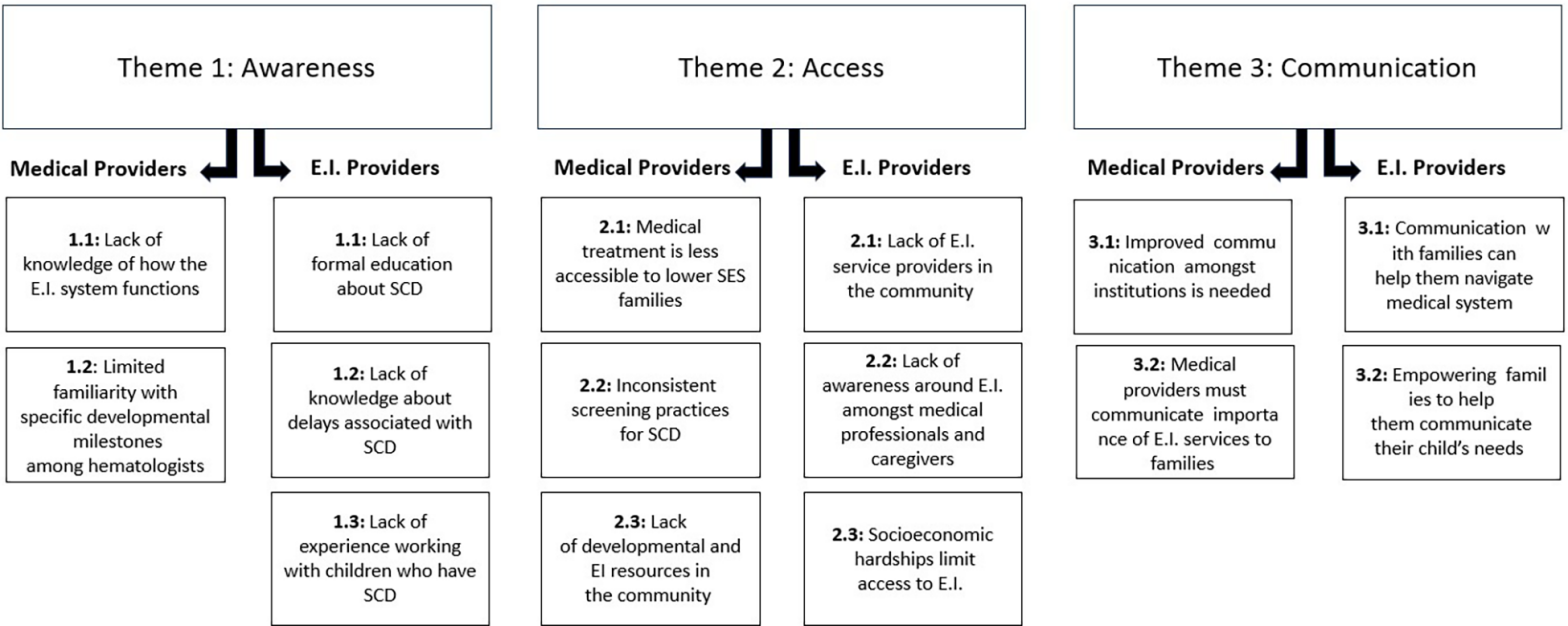
Interview themes and subthemes. This figure demonstrates the themes and subthemes that emerged from analysis of interview data. The themes of Awareness, Access, and Communication were shared across all participants, but content differed between medical and early intervention providers.

While some concepts were unique to medical providers and EI providers, the study team identified three overarching themes from both groups: Awareness, Access, and Communication (see Tables 1 and 2). These themes broadly aligned with multiple domains from our initial RE-AIM framework. Awareness encompassed Reach and Adoption, Access covered Reach and Implementation, and Communication involved Reach, Effectiveness, and Implementation. Although these three themes were shared by both medical providers and EI providers, the differing perspectives of each produced subthemes unique to the two professional fields (Figure 1).

**Table 1:** Medical providers subthemes and quotes.

	Theme	Subthemes and illustrative quotes
1	Awareness	1.1: Lack of knowledge of how the EI system functions Quote: “*MP-05: I… no. I guess I mentioned it already, I think. But just and I’m sure you guys are working towards this provider having a better understanding of what is actually done when we identify something, because I don't really know what goes on in the [EI services] program or any kind of program like that. So I think knowing what it looks like would help us kind of better sell it to the patients*. 1.2: Limited familiarity with specifics of developmental milestones among hematologists Quote: *MP-05: I mean, in theory, we would love the PCPs to be picking up on this because they are the driving force of the developmental assessments, and you know, being able to, to look at that because we are supposed to, in theory, be their hematologist, so we address their sickle cell concerns.*
2	Access	2.1: Medical and EI treatment is less accessible to lower SES families Quote: *MP-05: Yeah. Um, I mean, a lot of, a lot of my families, I’ve got single parents. I’ve got single parents with multiple kids, childcare issues, parents need to work. They can't be home for TEIS to come see them or get them to speech therapy at 3 o’clock when they don't get off until 6:00… they have to prioritize the things in their world which mean the most, which is keep the roof over their head and food in the house. And so that can be a huge challenge when we are dealing with, um, not a tremendous amount of support*. 2.2: Lack of developmental and EI resources in the community Quote: *MP-03: Well, that makes a lot of sense. I wonder how much, how, I guess, how much of, if everyone qualifies, are there enough services out there?* 2.3: Inconsistent screening practices for SCD Quote*: MP-02: I think if there were some screening tools, which, uh, most of the hematologists feel like if we had something that was more concise, more adapted for our patient population and something that can be done a little bit like pretty sensitive, maybe not that specific, but which can identify there's something going on. Uh, if that we can do ourselves and I think that would be the ideal situation.*
3	Communication	3.1: Improved communication among institutions Quote: *MP-05: So there was not a good communication system in play for when they get the referral, but then we don't find out what they do with that…if I were looking into the ideal world where we could build some more relationships as directly with, um, the TEIS program um and their counterparts in the other cities, um, to be able to have better communication, and to know who we're working with*. 3.2: Medical providers must communicate importance of EI services to families Quote: *MP-02: I don't think [caregivers] know much [about associated delays]. I think it's only when somebody has to go through that they're educated, but if they've had other kids with this issue, yes, then they would know something, or if they've had family members who've required these services, then they have some idea. But for somebody who this is their first time, I don't think it's like really well known unless their school identifies it and the school tells them about it.*

**Table 2:** Early intervention providers subthemes and quotes.

	Theme	Subthemes and illustrative quotes
1	Awareness	1.1: Lack of knowledge about delays associated with SCD Quote: *ICTS.EI.01: And then if I were to say more specifically for sickle cell, … that's not something that is necessarily like hot or like on my radar to like look out for a kid that has sickle cell to be like evaluating them*. 1.2: Lack of formal education about SCD Quote*:* *EI-05: Um. So I think any sort of education that we as early interventionists could receive prior to um treating kids with sickle cell would probably be um incredibly beneficial*. 1.3: Lack of experience working with children who have SCD Quote*: EI_04: Uh I really have not had that many kiddos with sickle cell. I've been working 12 years in early intervention um and I think I've had two patients the whole entire time.*
2	Access	2.1: Lack of EI service providers in the community Quote*: EI-01: Number one is location. So… and this is something that I can tell you based on, like, the clinic side, but also that I want to mention, if you’re in a location where there's no therapist that serve that area or not enough therapists, you’re not going to get services*. 2.2: Lack of awareness around EI amongst medical professionals and caregivers Quote*: EI-03: oftentimes I think when a parent brings up initial concerns, a lot of um medical staff are just very much with the mindset of let's just wait and see, wait and see. And so I would suggest that they were a little bit quicker to just refer to the people that do specialize in early childhood development and that way that parent would be able to get oriented to it*. 2.3: Socioeconomic hardships limit access to EI Quote: *EI-02: some people who have access to transportation and time will drive 45 min to Jackson where there's pediatricians, but again, if you’re a low-income family or a minority family who doesn't speak English and you’re going just to the clinic, the walk-in clinic every time your kid gets sick.*
3	Communication	3.1: Communication with families can help them navigate the medical and EI system Quote: *ICTS.EI.06: If we send information in a way that I think that that just doesn't generalize everybody in terms of getting the information, explaining every family are a different level. And sometimes we tend to generalize, information we disseminate*. 3.2: Empowering families to help them communicate their child's needs Quote: *ICTS.EI.02: Well, essentially, the biggest benefit is just helping parents understanding their role as educator to the children and helping them understand what they need to be doing so that because we're only there for an hour, a week. So working with the parents and saying, hey, you're the biggest you're the kid's biggest teacher. This is what you can do to ensure that your child is, you know, working hard to catch up on their milestones.*

## Awareness

### Medical providers

Providers were transparent about only having a basic knowledge of the Early Intervention Service referral process beyond their role in the initial referral. They recognized the need for a better follow up system by which they could track whether families were successfully connected to services.

*MP-05: I don't really know what goes on in the [EI services] program or any kind of program like that. So I think knowing what it looks like would help us kind of better sell it to the patients.*

Another provider had a general understanding of how the program is meant to operate but was unsure of how the process looks beyond the initial screening.

*MP-03: My, my understanding is once they're referred, there's a battery of evaluations that occur to see what services they need, whether it's speech, OT, PT, and then they're plugged in to those services. And then, that's the extent of what I know… I probably should know more about what exactly they do because I don't… I don't really know the process, so that would be helpful to know.*

The medical providers recruited for the Memphis study site work predominantly in a hematology setting. Along with confusion regarding the process for EI services, some providers expressed concern regarding their knowledge of development and their ability to identify smaller delays due to their specialized training and experience. One provider noted that the delays she identifies are usually marked as serious delays.

*MP-02: Um, I think the ones that we, (laughs) that I can catch are the ones that are pretty obvious that they have some like, you know, um gross motor delays or like verbal, … they require speech or something like that like something that's very obvious that they've not attained the milestones.*

Hematology practitioners report preferring primary care pediatricians (PCP) to survey for developmental delays and learning difficulties for this reason. PCPs monitor developmental milestones as part of regular checkups, so specialty practitioners feel it may be more efficient to have PCPs be the driver of identifying developmental delays and referring to EI services. In contrast, Hematologists do not give full developmental evaluations in their appointments, contributing to minor delays going unnoticed.

*MP-05: I mean, in theory, we would love the PCPs to be picking up on this because they are the driving force of the developmental assessments, and you know, being able to, to look at that because we are supposed to, in theory, be their hematologist, so we address their sickle cell concerns.*

Furthermore, hematology practitioners' focus is ensuring the child's SCD is well-managed and developmental delays are outside of their scope.

*MP-03: But we definitely advocate for the PCP because we're not it. We're looking at your blood, and that's what we're taking care of.*

Hematology practitioners' thoughts regarding who should drive identifying developmental delays were supported by primary care physicians (PCPs) interviewed in the St. Louis catchment area. PCPs reported conducting developmental surveillance regularly at wellness visits using measures such as the Ages and Stages questionnaire and the Modified Checklist for Autism in Toddlers.

*MP-06: Well for every patient single one of our patients, they get an Ages and Stages Questionnaire at … 9 months, 18 months, and 30 months. We do that standardized, and then we do MCHAT at 18 months and 24 months.*

PCPs had greater awareness of early intervention services and reported regularly referring children to services when they identified a developmental concern.

*MP-01: Well, if it's normal, nothing. If it's abnormal, I will use it. If they're under age three, I will refer them to [early intervention services] or early intervention, depending on where they live. Um, if they're over age three, then I will determine if I feel like there's a specialist specifically like PT, OT, or speech. And then I will refer them to that specific specialist at [location], or I will have the patient um contact the school district and get a full evaluation, including an Individualized Educational Plan and receive services through the school district.*

PCPs emphasized the importance of children with SCD attending their regularly scheduled well-child visits and hematology visits. They acknowledged the differing, yet essential, roles of hematologists and PCPs in providing care for patients with sickle cell disease.

*MP-07: Typically, I manage their [children with SCD] basic health care, annual checkups and developmental assessments and that sort of thing. And then the hematology specialist then takes care of the patient issues with regard to their sickle cell disease.*

While PCPs are more familiar with the process of obtaining EI services than hematology practitioners, they report a lack of follow-up from EI coordinators.

*MP-06: I would appreciate it if the coordinator would call me and let me know what the plan is and close the loop … They never do. The parents don't always know what they are getting. I don't receive any support.*

### EI providers

While medical providers reported a lack of familiarity with EI services, EI providers discussed lacking knowledge about SCD and the associated delays. All EI providers we spoke to discussed having very little formal education about sickle cell disease.

*EI-06: And then if I were to say more specifically for sickle cell, … that’s not something that is necessarily like hot or like on my radar to like look out for a kid that has sickle cell to be like evaluating them.*

In addition to lack of formal training about SCD, EI providers discussed having limited experience providing services to children with SCD.

*EI-01: I understand like, the pathology of it from grad school, but I don't think I've ever had a kid with diagnosed sickle cell disease throughout my entire career yet.*

*EI-04: I really have not had that many kiddos with sickle cell. I've been working 12 years in early intervention and I think I've had two patients the whole entire time.*

*EI-05: sure there are some [EI providers] that probably are [experienced in sickle cell], but I would say that the vast majority of us don't have much experience with it.*

EI providers expressed an interest in additional education and training, such as a continuing education course, to address this gap in knowledge and experience so they can better serve children with SCD.

*EI-04: I do think it'd be great if we could attend an in-service um on sickle cell disease and other blood disorders.*

*EI-05: So I think any sort of education that we as early interventionists could receive prior to treating kids with sickle cell would probably be um incredibly beneficial.*

Medical and EI providers indicated a need to improve knowledge and education around SCD and EI services from differing perspectives. Medical providers would benefit from information regarding when to refer to EI services and the post-referral process. EI providers would welcome educational opportunities on SCD to serve those children and families better.

## Accessibility

### Medical providers

Medical providers identified barriers to getting young children with SCD connected to EI services. Barriers included socioeconomic hardships for families, a lack of community resources to address developmental concerns, and inconsistent screening practices for developmental delays.

Regarding socioeconomic status (SES), medical providers acknowledged the obstacles that families with fewer financial resources navigate to get their child to medical visits. EI services are difficult to prioritize for families struggling with basic needs.

*MP-05: I've got single parents with multiple kids, childcare issues, parents need to work. They can't be home for TEIS [Tennessee Early Intervention System] to come see them or get them to speech therapy at 3 o'clock when they don't get off until 6:00… they have to prioritize the things in their world which mean the most, which is keep the roof over their head and food in the house. And so that can be a huge challenge when we are dealing with, not a tremendous amount of support.*

Providers discussed transportation as a barrier to services in addition to competing family needs. Multiple providers discussed the benefit of EI services potentially being in the home; an option that would eliminate transportation needs but would still require caregivers to take time off work. One provider suggested home appointments take place outside of traditional working hours to reduce the amount of time caregivers miss work.

*MP-02: I feel like one of the main reasons that a lot of appointments are missed uh is also because the parents are not able to get kids because of their work stuff. So, I think… if it can be done at home or if there's any home services or if it's something close to the community that they can access closer to home or if it's something after hours or weekend that they don't have to like, you know, uh like kind of miss work to do that, I feel like there'll be more adherence to it.*

Hematology providers expressed frustrations regarding inconsistent screening for developmental delays. They wanted a brief screening tool more suitable for children with SCD that they could administer in their clinics as well.

*MP-02: “think if there were some screening tools, which, uh, most of the hematologists feel like if we had something that was more concise, more adapted for our patient population and something that can be done a little bit like pretty sensitive, maybe not that specific, but which can identify there's something going on. Uh, if that we can do ourselves and I think that would be the ideal situation.*

This provider felt that this could address an additional barrier of long wait time to get SCD patients seen by psychologists or other developmental specialists.

*MP-03: A lot of times when we try to schedule the appointments for like psychology or like getting the educator involved just to do the screening, it never happens because, you know, they don't come back for those appointments and then things just like, you know, get delayed. So, if there is something more concise, a screening tool that the hematologist can be trained or like, the first line people, so the hematologist, the attendings or the advanced practitioners, can be trained in doing that might I feel speed up the process and instead of just referring to the next step.*

Long wait times were also discussed due to not having enough EI providers in the community. This was particularly a concern for providers in Tennessee, where SCD was recently added to the list of conditions that automatically qualifies a child for EI services. Although automatic qualification for EI services will improve access for patients with SCD, there may not be enough providers to meet the needs of Tennessee communities.

*MP-03: Well, that makes a lot of sense. I wonder how much, how, I guess, how much of, if everyone qualifies, are there enough services out there?*

### EI providers

Some EI providers expressed similar concerns about EI resources in the community, particularly a finite number of EI providers. EI providers complete intervention visits in the families' homes. Some providers who were predominantly in rural communities may travel up to 2 h between visits, which takes away from the number of children they can treat.

*EI-01: Number one is location. So… and this is something that I can tell you based on, like, the clinic side, but also that I want to mention, if you're in a location where there's no therapist that serve that area or not enough therapists, you're not going to get services.*

Another barrier identified by EI providers is the lack of EI awareness in the medical community. As noted in the education section, medical providers often lack understanding of EI services and may only refer for major delays. EI providers reported this as being a major barrier to getting children into services, as medical providers are the primary gatekeepers to obtaining additional services.

*EI-03: Oftentimes I think when a parent brings up initial concerns, a lot of medical staff are just very much with the mindset of let's just wait and see, wait and see. And so, I would suggest that they were a little bit quicker to just refer to the people that do specialize in early childhood development and that way that parent would be able to get oriented to it.*

PCPs see patients at a higher frequency and are in a better position to identify delays and when a child needs services. While families can access services without a referral from a physician, ensuring that pediatricians are aware of EI services and when to refer children is an essential step towards addressing this barrier.

*EI-08: Making sure my pediatricians are aware of the programs. And not only that, they are aware… you provide brochures for the office, for parents, you know, for children who has you know, these conditions….*

*ICTS.EI.01: The biggest barrier to providing services to them would, I mean, probably be even identifying them at all. So like, you know, we can't provide services for kids that we don't even have on our radar. So I think that's probably the biggest barrier is just that if those kids aren't getting referred to our early intervention programs, then we don't even know that they're out there or that they need assistance to initiate services and to provide those resources.*

This barrier is further exacerbated by families residing in rural areas, who may not see a pediatrician but a family medicine doctor. Family medicine physicians have less specialized training in developmental milestones and are even less likely to refer children to EI services due to lack of identification.

*EI-02: I feel like a lot of our kids don't go to pediatricians, they go to the family doctors and nurse practitioners in rural areas. And there's not a lot of education, because they're seeing from birth to your grandmother. I don't think they really know how quickly to respond to a developmental delay or what condition, you know, they're great at the medical management of these. And when do we refer to [children's hospital], [University hospital], [specialist children's hospital]… But knowing things like oh, we should get therapy started before they start school. There's… there's just not that…*

In addition to medical providers' lack of awareness of EI programming, families being unaware of EI services hinders access to services. EI providers specifically discussed families needing to know that these services exist and that the services primarily occur in the families' homes.

*EI-05: I think sometimes it's just a lack of knowledge, that early intervention services exist. that you know, they don't- the parents don't have to take the child anywhere.*

Families of children with SCD often must travel to multiple medical appointments and are uneasy about adding an additional clinic visit to their schedule. Home visits reduce the burden of lack of transportation.

It can be difficult to get a physician to refer for a developmental delay, and many families do not know they do not need a physician referral to be evaluated for EI. However, they do need a referral or an evaluation to get into services.

*EI-05: a lot of families don't understand, and doctors for that matter, that you don't have to be a doctor to make a referral for early intervention services. I can call as a parent and say, “Hey, I'd like to have my child evaluated,” and they're going to come out and do it. and a lot of people don't understand that they think it has to come from a doctor or a nurse or someone medical.*

Increasing awareness around the various avenues to obtain EI services will empower families to help their children access care. It is important to improve families' knowledge of developmental milestones in tandem, so they are familiar with both how to obtain services and when services are needed.

EI providers expressed similar concerns regarding SES and resources hampering families' access to EI services. Although EI services take place in the home, transportation was still cited as a barrier to accessing EI services, particularly for families in rural communities. Lower income families are less likely to travel over an hour to a city with pediatric providers.

*EI-02: some people who have access to transportation and time will drive 45 min to Jackson where there's pediatricians, but again, if you're a low-income family or a minority family who doesn't speak English and you're going just to the clinic, the walk-in clinic every time your kid gets sick.*

As previously noted, family medicine providers are not as skilled at identifying developmental delays and referring to services, and walk-in clinics are not screening for developmental delays. When families do overcome the issue of being referred to and obtaining services, some caregivers struggle to be available for the home visits. Caregivers may not be able to take time off from work. Others may be navigating their own chronic illness or mental health condition.

*EI-08: So it’s not necessarily that lack of transportation is a big barrier for a lot of our families, but not just like the lack of transportation, but the lack of like okay, a caregiver to attend appointments or to attend meetings and sit in and, you know, really be fully involved, whether it’s due to an, you know, employment or lack thereof or mental health concerns like because that that can also be a barrier to the parent has like a disability, you know, of their own.*

Medical and EI providers had overlapping concerns regarding barriers to accessing socioeconomic status, albeit from different perspectives. SES was frequently noted as a major factor for families’ inability to utilize EI resources. In addition to SES, both providers expressed the lack of EI services in the community contributes to difficulty obtaining services.

## Communication

### Medical providers

Communication and linkages among institutions (e.g., general pediatricians, EI providers, hematology teams) need to improve to better serve children with SCD who need EI services.

*MP-05: So there was not a good communication system in play for when they get the referral, but then we don't find out what they do with that…if I were looking into the ideal world where we could build some more relationships as directly with, the TEIS program um and their counterparts in the other cities to be able to have better communication, and to know who we're working with.*

Medical providers worry that some children get lost in the process due to this lack of communication between medical providers and EI providers. Building networks among pediatricians, hematology practitioners, EI providers and the community is necessary to prevent families from falling through the cracks.

*MP-03: “I would say we’re maybe building relationships between the providers here and the community to, to have a safety net for communication and follow-up. I think that would, that would help because if we're referring out, um, to a service, I mean, to a program and we don't really know what's going on, it's, I just think that some of those kids get lost… I definitely will say that there's definitely a positive impact, but I just don't know if we have good relationships to stay connected, I guess.”*

One provider suggested including developmental screening and EI services in the electronic medical record. Other medical processes have automatic triggers in the medical record to order consults or medications. It may be beneficial to have triggers for developmental evaluations or other EI services built into medical record systems for children with SCD.

*MP-9: Yeah, I mean, what would be amazing if it was maybe part of the electronic health record…e like those navigators in EPIC where you click, click, click and then, you know, if you click something that’s a trigger and triggers a psych consult or triggers, you know, other referrals.*

In addition to communicating across professions, medical providers need to communicate the importance of developmental services for children with SCD to caregivers in a manner that builds trust.

*MP-02: I don't think [caregivers] know much [about associated delays]. I think it's only when somebody has to go through that they're educated, but if they've had other kids with this issue, yes, then they would know something, or if they've had family members who've required these services, then they have some idea. But for somebody who this is their first time, I don't think it's like really well known unless their school identifies it and the school tells them about it.*

The trust component is essential to these conversations, as some caregivers might not be ready to accept their child might benefit from EI services.

### EI providers

EI providers expressed concerns that caregivers of children with SCD may not have the information needed to navigate the medical and EI systems and that current methods of providing information are not sufficient to help parents understand EI services. One provider explained the importance of conversing with caregivers to ensure their understanding of the EI referral process.

*EI-03: I ask who referred them for OT or why they thought that an OT evaluation was needed, they're like, “I don't really know. My doctor just kind of told me.” So, I'm like, “Okay you were just told you, you weren't explained. So, that's a little frustrating.*

*EI-03: I think like education, but when I say education, also like asking questions more than just handing them a piece of paper with this information because the reality is half of the time these parents don't even look at these handouts. Um, and I think it's very different when you sit one-on-one with them, even if it's five extra minutes.*

One provider mentioned meeting families where they are and tailoring information to each family to ensure they understand.

*EI-06: If we send information in a way that I think that that just doesn't generalize everybody in terms of getting the information, explaining every family are a different level. And sometimes we tend to generalize, information we disseminate.*

Families can be overwhelmed or scared when learning about potential developmental delays for their child. EI providers discussed the importance of empowering families to understand their role in helping their child improve.

*EI-07: Well, essentially, the biggest benefit is just helping parents understanding their role as educator to the children and helping them understand what they need to be doing so that because we're only there for an hour, a week. So working with the parents and saying, hey, you're the biggest you're the kid's biggest teacher. This is what you can do to ensure that your child is, you know, working hard to catch up on their milestones.*

Both medical providers and EI providers acknowledged a need for improved communication with caregivers. Medical providers spoke of their role in motivating parents to seek treatment by effectively communicating the importance of early intervention, while E.I. providers suggested ways to empower families to navigate the E.I. system. Medical providers also stressed the need for better communication between providers to improve efficiency.

## Discussion

SCD is a monogenic blood disorder characterized by progressive neurocognitive deficits and functional limitations. To address these deficits early in life, young children with SCD can benefit from intervention services. EI is a nationwide program that can improve neurodevelopmental outcomes for young children (11, 12), but the available evidence suggests that most patients with SCD do not receive these services (14). Through semi-structured interviews with medical and EI providers, we identified three broad themes that influenced EI utilization among young children with SCD: Awareness, Access, and Communication. These perspectives highlight next steps towards designing implementation strategies to improve EI access for this vulnerable population.

Both medical and EI providers described a lack of education and awareness to best serve the developmental needs of children diagnosed with SCD. Specifically, medical providers noted limited awareness about how the EI system works and familiarity with developmental milestones among hematologists who serve these patients. These observations suggest a need for more formal training and continuing education among pediatric hematology providers regarding screening for delays and referring for developmental services. It was noted that several pediatric hematology providers deferred to other disciplines (e.g., pediatricians, psychologists) for their expertise in these areas. Hematology practitioners reported preferring PCPs survey for developmental delays and learning difficulties. They noted PCPs monitor developmental milestones as part of regular checkups making it more efficient to have PCPs be the driver of identifying developmental delays and referring to EI services. Given this preference, it may be helpful to have PCPs embedded within SCD clinics to address these topics and provide education and awareness. Future research should examine ways in which children with SCD can remain engaged with primary care providers to ensure access to developmental screening and referrals. EI providers consistently expressed a lack of knowledge and awareness about SCD. EI providers had limited experience working with patients with SCD or learning about the developmental and medical risks associated with the disease. These providers expressed a strong interest in learning more about SCD. Given these gaps in knowledge and the desire to learn more, formal educational materials and activities should be developed for EI providers to help tailor appropriate services for patients with SCD. For example, EI providers could potentially detect if poor engagement in a session is due to experiencing pain or discomfort and learn behavioral strategies to respond to pain/discomfort and practice these strategies with caregivers. Further, EI providers could reinforce the importance of medical adherence with caregivers to limit medical and developmental complications associated with SCD.

Significant barriers limiting access to EI services were reported. Both medical and EI providers noted that families from lower socioeconomic status backgrounds had a harder time obtaining EI services and that there was a lack of EI resources in the community. Barriers to accessing these services included transportation, distance to providers, and lack of services offered after work hours. Even when EI services are offered in the home environment, providers shared that many parents are unable to take off work to attend. These barriers are consistent with parent-reported barriers among low-income minority families (20). EI providers shared that they frequently drive long distances for home visits, limiting the number of patients that they can serve. Potential solutions include increasing access to virtual/telehealth services, offering more services before or after work hours, or creating satellite centers in more rural areas. Several studies have suggested that low-income and rural families have had positive experiences with telehealth services for early intervention, although some additional barriers exist (e.g., internet access) (28, 29). A combination of these solutions may allow EI providers to serve more patients and improve access for families from lower socioeconomic backgrounds. In addition to difficulties accessing intervention services, medical providers noted that there were inconsistencies in screening and assessment for developmental delays in patients with SCD. They described long wait times to see a developmental specialist or psychologist to determine if EI services were needed. These concerns are exacerbated by a lack of knowledge about the EI system and determining the best route to funnel patients into services. Recent guidelines published by the American Society of Hematology suggest standards of care for developmental screening in children with SCD; however, it is unclear how these standards have been implemented across medical settings.

EI and medical providers described breakdowns in communication between providers and the information that is shared with families, limiting the utilization of EI services. Medical providers consistently discussed a lack of follow-up and communication from EI services. They noted that after they referred a patient, it was hard to determine if the patient had ever been seen or if the referral had even been received/processed. Many patients were “lost” in the system, and it was difficult for medical providers to determine who they should contact. Medical providers indicated that it was important to build relationships with EI providers in the community to have a safety net for patients if the initial referral was unsuccessful. From the perspective of EI providers, there were consistent concerns about families becoming “lost” in the system. EI providers stressed the importance of providing education to families about the EI system and how to obtain services. They also highlighted the need to empower families to help their child. EI providers noted that there are limitations to what they can accomplish with a child when they are only meeting with them an hour per week. Rather, EI and medical providers should strive to help parents understand their role as an educator and teacher. These differing perspectives highlight the need to build relationships between medical and EI providers to develop a common understanding of perceived roles and the best ways to facilitate each profession to be successful. The communication breakdown reported by EI and medical providers is consistent with prior research among low-income families, noting that they received conflicting information from different providers (20), limiting their desire to participate in EI services. To bridge the communication between these groups, families may benefit from a patient navigator to increase follow through with referrals (30).

This study has several strengths. To our knowledge, this is the first study to assess provider perspectives on EI utilization in SCD, and the collected data generated novel ways to improve developmental services for young children with SCD. Perspectives were collected from both medical and EI providers. To increase generalizability of the findings, data was collected across two states and hospital systems. Yet, several limitations do exist. All interviews were conducted virtually (over the phone or video-conference), potentially limiting comfort and rapport building with providers. Team members knew some of the participants in a professional capacity, potentially biasing their responses. Limited data were collected assessing provider demographics and background making it difficult to thoroughly describe the sample of participants.

## Conclusions

Patients with SCD are at high risk for developing developmental delays that worsen as they grow older. EI services early in life can limit these delays and improve long-term outcomes, yet most families of young children with SCD do not utilize these services. Semi-structured interviews with medical and EI providers identified three broad themes that influenced EI utilization: Awareness, Access, and Communication. These themes highlight numerous gaps that facilitate the testing of targeted solutions to increase EI utilization among this vulnerable population, such as (1) increasing provider education (e.g., handouts or didactics on SCD for EI providers), (2) improving access to services (e.g., via virtual/telehealth options), and (3) improved communication between providers (e.g., via patient navigator). Further research is needed to explore the feasibility and efficacy of these potential solutions.

## Data Availability

The raw data supporting the conclusions of this article will be made available by the authors, without undue reservation.
