# Supplementary material for: Awareness, access, and communication: provider perspectives on early intervention services for children with sickle cell disease
Source: Front Pediatr. 2024 Mar 25;12:1366522. doi: 10.3389/fped.2024.1366522 (PMC11000123; doi:10.3389/fped.2024.1366522)
Supplement: Supplementary file 1 [file Datasheet1.pdf]

Interview guide for Early Intervention Providers of Children with SCD

Intro:

Thank you for speaking with me today about your experiences providing early intervention services. We appreciate your time, and we look forward to your input. The purpose of this interview is to understand early intervention services for young children diagnosed with sickle cell disease. ***There are no right or wrong answers—it is your observations and opinions that we are interested in.***

The interview will be recorded and should take about 30 minutes. If you have any questions throughout or after the interview, please let me know. We can stop the interview at any point, if necessary.

I will start by asking some general questions, and then I will go over other more specific questions. Do you have any questions before we begin?

Section 1: General:

Data goal: who are you, what do you know about SCD?

Education for all providers: Sickle cell disease is a genetic blood disorder affecting approximately 100,000 individuals in the United States. Black individuals experience the highest prevalence rates. The disease distorts the shape of hemoglobin molecules, which transport oxygen throughout the body. Symptoms include anemia, infection, pain crises, stroke, tissue damage, organ failure, and premature death. Patients frequently experience developmental and learning difficulties such as problems with speech or learning academic concepts.

1) What is your job title and/or role?
2) Describe your experience providing and/or facilitating early intervention services.
3) What do you know about sickle cell disease?
4) Based on your experience, information from the medical team, and/or your own research what is your understanding of developmental or learning difficulties children with sickle cell disease might experience?

Section 2: Reach:

Data Goal: Perceived barriers for EI for SCD

1) Have you ever worked with a child with sickle cell disease?
    a) **Prompt, if yes**: what did that experience entail?
    b) **If no,** why not? and go to #2.

2) What barriers prevent children and their families from obtaining early intervention services?
    a) **Probe:** What are the requirements for a child *[with sickle cell]* to qualify for services?
    b) **Probe:** For early intervention, what services are available to participating families and children?
        i) Where are services provided? Are transportation services offered?
    c) **Probe:** What support services (apart from direct therapies) are there available families?
        i) Support groups? Sickle Cell specific support? Parent support?
    d) **Probe:** The majority of children with SCD identify as Black or African American. What programming or structural support exists to facilitate engagement in early intervention? Are there measures taken into consideration to deal with the racial and cultural diversity in the patient population?
    e) **Probe:** Certain medical conditions automatically qualify for early intervention services. How are you involved in deciding what conditions or children qualify?

Section 3: Effectiveness:

Data Goal: Perceived benefits of EI for children with SCD

1) Do you find early intervention services to be effective in improving developmental outcomes in young children?
    a. **Probe**: What benefits do services provide?
    b. **Probe**: What changes do you typically see?
    c. **Probe**: What involvement do parents have in the intervention process? *What does it look like?*
2) [*Based on your understanding of sickle cell disease*], how would young children with sickle cell disease benefit from early intervention services?
3) What suggestions do you have for improving and making easier the process of obtaining early intervention services for patients with sickle cell disease and their families?

Probe: What do you think the “ideal” therapy/intervention for SCD would look like?

Section 4: Adoption:

Data Goal: Strategies to prepare EI providers to treat children with SCD

1) What do you think EI providers need to be able to support and treat children with SCD?
    a. **Probe:** Are most early intervention providers familiar with sickle cell disease and the associated learning and developmental difficulties?

2) What factors might interfere with your team’s ability to provide early intervention services to children with sickle cell disease?

Section 5: Implementation:

Data Goal: Strategies to get EI providers on board with making kids with SCD eligible for EI

1) What would be required to provide early intervention resources to more children with sickle cell disease?
    a. **Probe:** Who would need them? How often and what format?
    b. **Refer to previous barriers the person described and ask how to fix them**

2) If you were to develop a program to provide intervention services for children with sickle cell disease what it look like?
    a. **Probe**: At what age would the services occur?
    b. **Probe**: Where would the services occur?
    c. **Probe**: How often would the services be provided?
    d. **Probe:** How would a patient with sickle cell disease qualify for services?
