# Supplementary material for: Awareness, access, and communication: provider perspectives on early intervention services for children with sickle cell disease
Source: Front Pediatr. 2024 Mar 25;12:1366522. doi: 10.3389/fped.2024.1366522 (PMC11000123; doi:10.3389/fped.2024.1366522)
Supplement: Supplementary file 2 [file Datasheet2.pdf]

Provider Interview Guide

Session Information

Interview date:
Provider ID number:

INTERVIEW GUIDES: MEDICAL PROVIDER

Thanks for participating in this interview. We appreciate your time and we look forward to your input. The purpose of this interview is to understand challenges to and resources available for performing cognitive screenings of young children aged 0-3 years with sickle cell disease (SCD) and referring them to further services. Please help us understand your practice so we can move forward with strategies to implement screening and referral programs and improve health for individuals living with SCD. There are no right or wrong answers—it is your observations and opinions that we are interested in.

The interview will be recorded and should take about 30 minutes. If you have any questions throughout or after the interview,please let me know. We can stop the interview at any point, if necessary.

I will start by asking some general questions, and then I will go over other more specific questions. Do you have any questions before we begin?

Section 1. Self-intro & familiarity with SCD

- Tell me about your role with [organization name] and the patient population you work with
- To give us some general background about your practice, could you BRIEFLY talk about your experiences taking care for patients with SCD,
    - How many patients with SCD do you see annually?

Section 2. SCD specific questions

- How often do you refer children with SCD for developmental testing?
- Besides the challenges you have mentioned, are there **additional challenges** to screen children with SCD and connect them to ongoing services?

Section 3. Recommendations

- Now imagine that we have all the resources for children with SCD, what would the developmental screening and referral process look like?
    - What would be the role of the pediatrician/hematologist ? (Who should be the main driver for this program)
        - Are there other providers that would be important to the screening/referral process?
    - Are there any changes that could be made so that early intervention services, which are provided for children birth to 3 who have developmental delays or disabilities, could be better utlized among young children with SCD?

- Is there anything that we haven't talked about that you think would be important for the success of a developmental screening program at St. Jude?

Section 3: Early Intervention add on:

Reach

Goal data: how do kids with SCD get therapy, how could more get it

1. What challenges do children with SCD usually show to qualify them for referral for early intervention services?
   0. **Probe:** What delays, if any, have you observed in children with SCD?
2. Tell me about what ideal services to support children and families with SCD would look like
   0. **Probe**: What about rehabilitation or therapy services?
   1. **Probe**: How can (St. Jude or Wash U) increase access to intervention services for these patients?

Effectiveness

Goal data: does therapy work? what therapy would be best?

1. What impact do you think early intervention services (like caregiver counseling, occupational or speech therapy for child) would have on children with SCD?
   0. **Probe**: Are there any specific therapies you think children with SCD could benefit from? Why?
   1. Tell me about the impact early intervention services might have on caregivers or families.
   2. **Probe:** How can early intervention services address the developmental/learning difficulties seen in children with SCD?

Implementation

Goal data: how could we get therapy to more kids with SCD?

1. How could the process of referral and obtaining permissions for getting early intervention services be made easier and more effective?
2. Why do you think caregivers may not follow up on a referral to early intervention services?
3. Tell me about what the ideal system would look like if it were designed to make the process very easy to get early intervention?

Adoption

Goal data: what would it take to get kids with SCD to use therapy services?

1. What is your understanding of early intervention services?
2. How do you typically address developmental concerns for your patients under three years of age?
3. To what extent is the local community aware of the developmental and learning difficulties observed in patients with sickle cell disease?

Maintenance

Goal data: if people know about delays in SCD, will they do something about it?

1. Now that we've had this conversation, will it impact your practice? If so, how?
